# Late–onset systemic lupus erythematosus-associated primary biliary cirrhosis

**DOI:** 10.1186/1755-7682-6-3

**Published:** 2013-02-05

**Authors:** Sonia Hammami, Nabil Chaabane, Houda Mahmoudi, Fethia Bdioui, Hamouda Saffar

**Affiliations:** 1 Department of Internal Medicine, CHU. F. Bourguiba, University Hospital F. Bourguiba, University of Monastir, Monastir, Tunisia; 2 Department of Gastroenterology CHU. F. Bourguiba, Monastir, Tunisia; 3 Department of pathology. CHU. F. Bourguiba, Monastir, Tunisia

**Keywords:** Systemic lupus erythematosus, Primary biliary cirrhosis, Antimitochondrial antibodies

## Abstract

**Background:**

The development of Primary Biliary Cirrhosis (PBC) during the course of Systemic Lupus Erythematosus (SLE) is extremely rare. We report the case of a geriatric woman who was diagnosed with SLE at 69 years of age then with primary biliary cirrhosis one year later.

**Case presentation:**

A 70-years-old woman, who had been diagnosed with SLE at 69 years, was admitted for further examination of liver dysfunction. PBC was confirmed based on elevated serum levels of transaminase, high levels of antimitochondrial antibodies and following a liver biopsy. The oral administration of ursodeoxycholic acid stabilized the liver dysfunction.

**Conclusion:**

We described an original case report of elderly patient with coexisting PBC and SLE. To date, according to the best of our knowledge, there have been few case reports of SLE/PBC co-occurrence. The aetiology of this complex remains unknown, autoimmune mechanisms, environmental and genetic factors are considered important in the susceptibility to both diseases. Osteopontin might play an important role.

## Background

Systemic Lupus Erythematous (SLE) is an auto-immune disease that occurs mainly in young women. Late-onset SLE has generally be defined as SLE onset after the age of 50 years. This later age at onset has a strong modifying effect on the clinical presentation [1]. Co-occurrence SLE and primary biliary cirrhosis (PBC) is rare. The development of PBC during the course of SLE is extremely rare [2]. We report, here a geriatric woman who was diagnosed with systemic lupus erythematosus at 69 years of age then with primary biliary cirrhosis one year later.

## Case presentation

**Figure 1:**
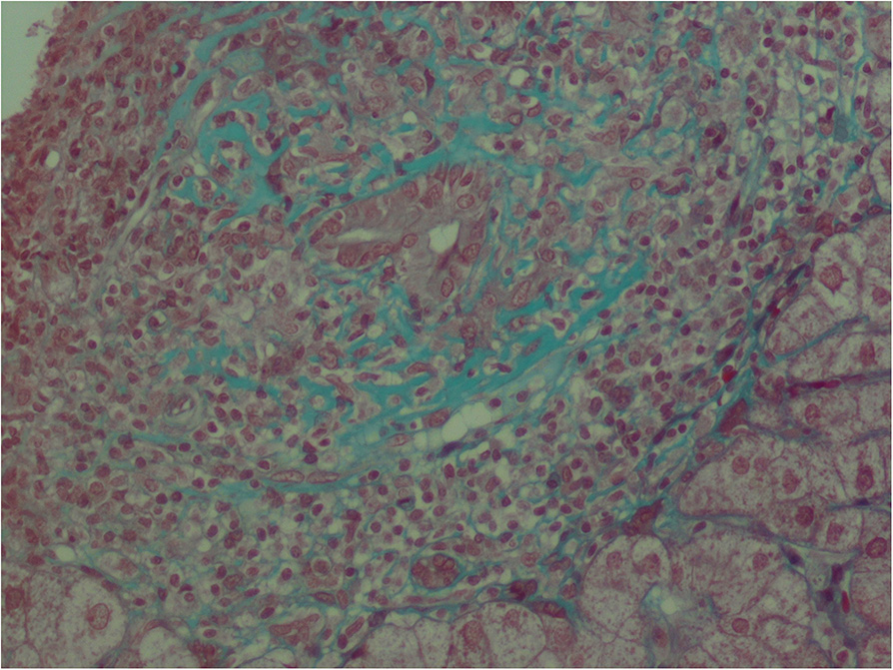
Florid duct lesions (Trichrome x400).

**Figure 2:**
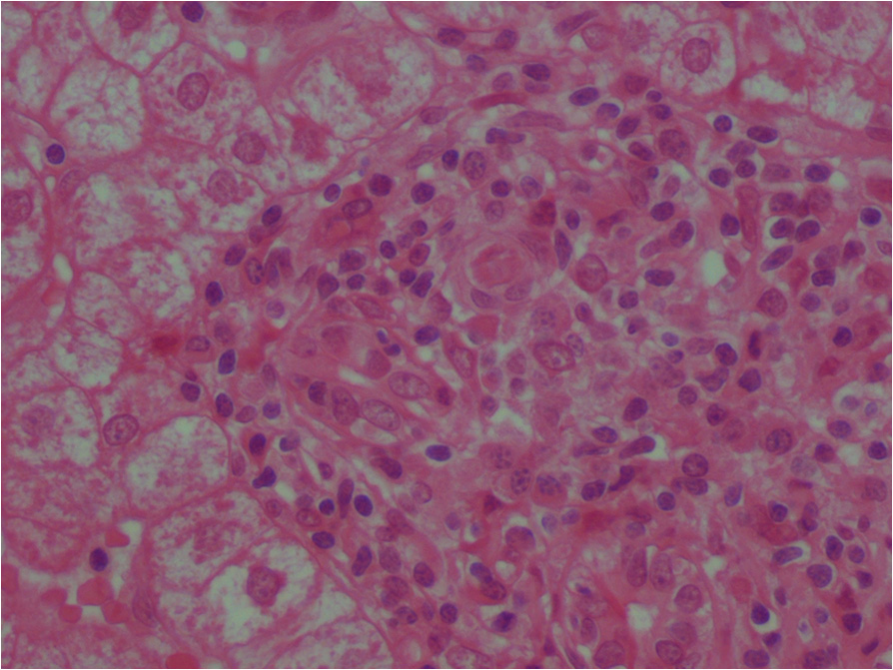
Lymphocytic cholangitis with plasma cells (HEx400).

A 69-year-old Tunisian female with no family history of auto-immune disease. She had a history of hypertension and was explored 4 years prior to admission for transient arthralgias and arthritis. At age 69, she experienced photosensitivity, malar rash and diffuse discoid lesions in her trunk and face for which she visited our hospital. She also reported weight loss of approximately 3 kg in the last three months. She had no history of drug abuse or significant alcohol consumption prior her admission. The physical examination showed synovitis of the wrists. Her general state of health was regular, eupneic. Cardiovascular and respiratory examinations were within the normal range. The patient had no previous laboratory tests. On admission, her initial laboratory tests were as follows: erythrocyte sedimentation rate at 45 mm/1st hour associated with hyper-γ-globulinemia 20 gr/l. Her leukogram showed lymphopenia 850/mm^3^, and platelets were normal. Creatinin and urinalysis are normal. The autoimmune profile confirmed a strong positivity of Antinuclear antibodies (ANA) with titre 1:400 and anti-double-stranded DNA (anti-dsDNA) at 115 UI/ml with normal serum complements. The patient was non-reactive for the following antibodies: anti-La; anti-cardiolipin; lupus anticoagulant; anti-SM; anti-RNP; anti-SCL-70; and anticentromere, rheumatoid factor and autoantibodies against citrullinated protein. Serology for VDRL was negative, hepatobiliary enzyme and lactate dehydrogenase levels were within normal range. Therefore, presence of 6 of 11 American College of Rheumatology criteria (photosensitivity + malar erythema + positive ANA and anti-dsDNA tests + hematologic abnormalities including lymphopenia + arthritis) allowed the diagnosis of SLE. The patient was treated with Chloroquine (200 mg/day). The patient showed improvement of her general state of health, and weight gain. No aggravation of SLE was observed. However one year later, she developed liver dysfunction. The patient was referred to gastroenterology unit. On physical exam, skin rash involving her face was observed. Abdominal examination revealed neither hepatosplenomegaly nor pruritus. Laboratory investigations revealed a normal complete blood count and urinalysis. His erythrocyte sedimentation rate was at 60 mm/1st hour while C-reactive protein was within normal range. Concentrations of both serum cholesterol and triglyceride are normal. Blood creatinine clearance 80 mL/min and there was no proteinuria. Aspartate aminotransferase 92 IU/L (normal range: 8–38); alanine aminotransferase 87 IU/L (normal range: 4–44 IU/L) (2 times above the normal limit), total bilirubin 0.9 mg/dl; alkaline phosphatase 107 IU/L, gammaglutamyl transpeptidase 42 IU/L and lactate dehydrogenase 230 IU/L are at normal range. Antimitochondrial antibodies (AMA) were positive (1:164), with positive anti-E2 fraction. The antinuclear antibody was positif as well as anti-dsDNA. Negative results were seen for anti-smooth muscle antibody and anti-centromere antibody. Serologic makers for hepatitis B and hepatitis C viruses were negative. Abdominal echo showed no fatty change of the liver and no abnormality of the bile ducts. Biochemical liver tests and AMA results are compatible to PBC. However, liver biopsy is required to give information on the stage of PBC. Microscopic examination reveals a focal florid duct lesion characterized by a portal lymphocytic infiltrate and epitheloid cells that are centred on interlobular ducts with evidence of destruction (Figure 1). These lesions are associated with a lymphocytic cholangitis and a portal inflammation containing conspicuous plasma cells and numerous lymphocytes (Figure 2). No granuloma was found in lobule. These findings led to a diagnosis of stage I PBC according to Scheuer’s classification. Based on the hematology and liver-tissue results, as well, she was diagnosed with asymptomatic PBC. Treatment with ursodeoxycholic acid (600 mg daily) was started. The transaminase levels normalized with one month. The patient has not complained liver dysfunction. She was followed up regularly with stable health.

## Discussion

Primary biliary cirrhosis is a chronic cholestatic liver disease of unknown aetiology characterized by immune-mediated chronic non suppurative cholangitis that mainly affects interlobular and septal bile ducts that eventually leads to cirrhosis and liver failure. Women are mainly affected, with a female/male ratio of 9/1 [3]. The median age of disease onset is 50 years. Half of the patients are asymptomatic at diagnosis. Fatigue and pruritus are the two symptoms of the early phase of the disease. PBC is associated with several extrahepatic autoimmune diseases including Sjögren syndrome, chronic thyroiditis and scleroderma [4,5], however, an association with SLE is not widely recognized. In most SLE/PBC coccurence cases, PBC develops before SLE. This in extremely rare case of a diagnosis of SLE followed one year later. There are only five such definite associations reported in the literature [2,6,7]. To the best of our knowledge; this is the first description of a geriatric patient (Table 1).

**Table 1:** Summary of primary biliary cirrhosis developed after systemic lupus erythematosus reported in the literature

**N**^**o**^	**Author**	**Sex**	**Age diagnosis of PBC**	**Age diagnosis of SLE**	**Symptoms**
1	Nishi et al. ^(in2)^	F	47	39	Jaundice
2	Michel et al.^(6)^	F	72	54	Jaundice
3	Matsuo et al.^(in2)^	F	52	46	Asymptomatic
4	Sato et al.^(7)^	F	44	27	Liver dysfunction
5	Shizuma et al.^(2)^	F	29	21	Asymptomatic
6	Our case	F	70	69	Asymptomatic

Late onset SLE patients are relatively rare, as most cases of SLE are younger female. Incidence of late onset SLE is 12–18% [8]. In elderly, SLE has been reported to have a clinical presentation different from that in younger individuals. Age modifies the clinical expression of the disease in terms of clinical and laboratory findings, pattern of organs involvement and severity of disease. The prevalence of serositis, lung involvement and Sjögren’s syndrome were observed more often. The prognosis in older patients is relatively good [9]. In our case, no aggravation of SLE was observed with the administration of chloroquine. In older SLE patients, the management of the disease depends on the type and severity of disease manifestations. The use of antimalarial agents is an important aspect of SLE treatment. Corticosteroid and immunosuppressive agents depending on which organs are involved. In severe cases of SLE, biological drugs targeting specific pathways (T-B lymphocytes interactions, cytokines and complements) have been proposed as new therapeutic approach for SLE [10]. This is especially important for early-onset SLE patients, in elderly patients, the less aggression clinical evolution, comorbidities and concomitant therapies may limit the therapeutic options for SLE [8,9].

The frequency of liver dysfunction during the course of lupus is high, in one retrospective study of 242 SLE patients, and liver enzyme abnormalities were observed in 45 patients (18.6%). Of these, only 14 cases (5.8%) could be attributed to lupus hepatitis [11]. Recently, Efe et al. reported in retrospective analysis of 147 SLE, that 36 (24.4%) of them had liver enzyme abnormalities, autoimmune liver disease who was diagnosed only in 4.7% of all SLE [12].

Liver dysfunction accompanying SLE correlating strongly with the presence of antibody to ribosomal P protein and may be due to steatosis from lupus or from corticosteroid therapy. Only about 10% of patients with autoimmune hepatitis have lupus [13].

In our patient, there are no histories of drug abuse, nor alcohol or toxic substance addiction and negative serologies for hepatitis B and hepatitis C viruses eliminated potential viral causes. In liver biopsy specimens, inflammatory cell infiltration, abnormal bile ducts in the portal areas hepatic granulomas in the hepatic lobules and the detection of AMA with positive anti E2 fraction were consistent with PBC diagnosis. Antimitochondrial antibodies M2 are very sensitive and specific serological marker of PBC. The E2 component of the pyruvate dehydrogenase complex and other members of the 2-oxo-acid dehydrogenase complex (2-OADC) are their molecular targets [3,14].

Our observation highlighted the importance of liver function evaluation in first assessment and during follow-up of SLE, even in the absence of symptoms (jaundice, pruritus, fatigue…), because clinical presentation is sometimes not obvious and the diagnosis can only be suspected by liver dysfunction.

Systemic lupus erythematosus (SLE) and primary biliary cirrhosis (PBC) are two autoimmune disorders. Although the autoimmune mechanisms behind the PBC/SLE association are not fully understood because there are few reported cases of both diseases. The pathogenesis of either disease has not yet been clarified, environmental and genetic factors are considered important in the susceptibility to both diseases [6,15]. Early T-lymphocyte activation 1 (Eta-1)/osteopontin (OPN) is a soluble ligand with pleomorphic immunologic activities which plays an important role in inflammation and immunity and may also contribute to the association between PBC and SLE [2]. OPN was reported to be highly expressed in the MRL/lpr mouse, which is recognized as one of the spontaneous autoimmune models of SLE. Recently Han et al. in a large multiethnic cohort: 1141 unrelated SLE patients [707 European-American, 434 African-American] confirm the association between OPN and SLE [16]. OPN may be involved in the susceptibility to PBC. It was shown that OPN is involved as a chemoattractant cytokine in the recruitment of macrophages and T lymphocytes in the liver granulomas in PBC [17].

## Conclusions

We have reported an elderly patient with SLE associated with PBC. It is unlikely that these diseases developed casually in our patient, considering the underlying autoimmune pathogenesis that they share in common. OPN might play an important role in both diseases.

## Consent

Written informed consent was obtained from the patient for publication of this case report.

## Abbreviations

SLE: Systemic lupus erythematosus; PBC: Primary biliary cirrhosis; anti-dsDNA: Anti-double-stranded DNA; ANA: Antinuclear antibodies; AMA: Antimitochondrial antibody; OPN: Osteopontin.

## Competing interests

The authors declare that they have no competing interests.

## Authors’ contributions

SH carried out the literature search and drafted the manuscript and given the final approval of the version to be published; NC collected the information regarding the case; HM is the pathologist; FB contributed to writing the manuscript did the critical revision for important intellectual content in the manuscript; HS was a contributor in writing the manuscript and co-authored the manuscript. All authors read and approved the final manuscript.
